# Noninvasive detection and prediction method for temperature in *ex vivo* biological tissue based on opto-thermal-acoustic co-coupling

**DOI:** 10.1117/1.JBO.31.5.057001

**Published:** 2026-05-28

**Authors:** Yuelin Han, Han Gong, Yiming Ma, Mingjian Sun

**Affiliations:** a Harbin Institute of Technology, Department of Control Science and Engineering, Weihai, China; b Harbin Institute of Technology Suzhou Research Institute, Suzhou, China; c Harbin Institute of Technology, Department of Control Science and Engineering, Harbin, China; d Harbin Institute of Technology (Weihai) Qingdao Innovation and Development Base, Qingdao, China

**Keywords:** multiphysics modeling, photoacoustic effect, photothermal therapy, thermometry

## Abstract

**Significance:**

Photothermal therapy is a minimally invasive technique that utilizes near-infrared light to induce localized hyperthermia for the selective ablation of cancer cells. Its therapeutic efficacy is highly dependent on precise temperature regulation. Aiming at the problems of invasiveness, insufficient penetration depth existing in current temperature monitoring technologies, we propose an innovative solution.

**Aim:**

We propose a method that can be used to monitor the temperature inside *ex vivo* biological tissues during laser heating.

**Approach:**

By constructing an optothermal-acoustic multiphysics coupling model and combining it with our designed dual-optical path co-coupled photoacoustic-photothermal temperature monitoring system, noninvasive observation of the internal heat source distribution in tissues is achieved. On this basis, theoretical data and real data are fused to realize precision measurement of the temperature in the lesion area and adjacent tissues.

**Result:**

The results of *ex vivo* experiments show that this method achieves heat source intensity estimation with an error of less than 3.5% in *ex vivo* tissues at the millimeter to centimeter depth range, and the temperature measurement error at multiple different validation sampling points is within 0.25°C.

**Conclusion:**

The proposed method and system in this study can calculate the temperature inside *ex vivo* tissues during laser heating, offering a broader temperature measurement range compared with conventional photoacoustic thermometry. This method possesses certain academic value and is expected to provide technical reference for the research on target temperature sensing during photothermal treatment.

## 1 Introduction

Photothermal therapy (PTT) is an emerging minimally invasive treatment technology. Based on the difference in heat tolerance between cancer cells and normal tissues, this technology utilizes near-infrared light to irradiate tissues and generate localized hyperthermia, thereby selectively killing cancer cells while minimizing damage to normal tissues.^1^ It offers advantages such as precise targeting, high controllability, and the potential for synergy with chemotherapy and immunotherapy. Furthermore, this technology has also been applied in fields such as antibacterial therapy and dermatology.^2^^–^^4^ The therapeutic efficacy of PTT is highly dependent on precise temperature regulation, and its mechanism of action exhibits a distinct temperature-dependent profile. Local temperatures ranging from 42°C to 45°C can induce tumor cell apoptosis with minimal damage to adjacent normal tissues and favorable safety. Temperatures between 46°C and 50°C cause irreversible protein denaturation and membrane structural damage in tumor cells. Temperatures exceeding 50°C may lead to damage of normal tissues. An ideal treatment requires precise control of the temperature distribution both temporally and spatially, ensuring that the tumor region reaches the effective therapeutic temperature while avoiding the impact of heat diffusion on surrounding healthy tissues. This temperature sensitivity makes real-time temperature monitoring and intelligent temperature control systems crucial for enhancing the safety and effectiveness of PTT.^5^

In the academic community, extensive research has been conducted on the noninvasive temperature measurement within biological tissues. Traditional infrared thermal imaging^6^^–^^8^ enables noncontact measurement with relatively high temperature measurement accuracy, yet it suffers from a limited detection depth and inadequate capability for temperature monitoring of deep-seated tumors. Ultrasound (US) thermometry, which relies on signal time shifts and characteristic variations induced by temperature changes, allows deep tissue temperature detection with favorable real-time performance,^9^^–^^12^ but its measurement accuracy is unsatisfactory. Magnetic resonance thermal imaging (MRTI) offers excellent deep tissue imaging capability,^13^^,^^14^ but its high equipment cost and low temporal resolution make it difficult to meet the requirements of real-time control during PTT. Although each of the aforementioned thermometry methods has its own merits, their inherent limitations prevent them from matching the precision demands of current PTT. The lack of reliable thermometry approaches has forced traditional PTT to adopt a “trial-and-error strategy” in clinical practice, wherein laser parameters are repeatedly adjusted to observe temperature responses—this method is inefficient and carries considerable risks. To address this issue, some researchers have proposed closed-loop PTT methods by combining the photoacoustic (PA) effect^15^^–^^20^ with automatic control theory,^21^^–^^24^ which has reduced the cost of the trial-and-error process to a certain extent. The PA effect refers to the instantaneous expansion and contraction of the irradiated tissue region following pulsed laser illumination, which further triggers tissue vibration and generates ultrasonic waves. Studies have demonstrated that within the temperature range preserving biological activity, the intensity of the PA effect exhibits a linear relationship with tissue temperature. Beyond this biologically viable temperature range, parameters such as tissue optical properties alter, rendering PA thermometry inaccurate. For mild PTT, the temperature variation falls within the linear range of PA thermometry, enabling preliminary *in vivo* tissue temperature measurement based on the PA effect.^25^^–^^28^

Although PA thermometry has partially satisfied the requirements for precise PTT, it still presents limitations. The effective temperature measurement region of conventional PA thermometry is dependent on the tissue volume covered by local PA signals, which is typically much smaller than the volume of the core high-temperature region inside the tissue. Consequently, single-point temperature measurement often fails to fully capture the temperature distribution within the tissue, further preventing the analysis of temperature differences between lesion areas and healthy tissues, and making it difficult to evaluate therapeutic outcomes. The establishment of reliable thermodynamic models and temperature prediction methods can effectively resolve these problems, with four core contributions: first, it allows pretreatment simulation of temperature field distributions under different laser parameters, providing a scientific basis for treatment planning; second, it enables real-time prediction of temperature evolution during treatment to support proactive regulation; third, it integrates heterogeneous tissue parameters to realize personalized temperature management; fourth, it acquires more comprehensive target temperature information inside tissues through theoretical calculation and data fusion. However, most current studies in this field remain at the stage of numerical simulation.^29^^,^^30^

To address the aforementioned requirements and deficiencies in current research, the main innovation of this paper is the proposal of a specialized dual-optical path co-coupled PA-PTT temperature monitoring (DOPT) system, which achieves macroscopic coupling of the therapeutic light field and imaging light field through a customized optical path design. This system can observe the heat source in the target region via the PA effect in tissues before heating and tightly couples the PA effect and photothermal effect within tissues during PTT. Methodologically, based on the conditions provided by the DOPT system, this paper constructs a strongly coupled light transport-thermal conduction-acoustic propagation multiphysics model (LTU Model) inside biological tissues. The Kalman filter is employed to fuse multisource data from PA thermometry and the LTU Model, which not only improves the accuracy of PA thermometry but also extends the temperature observation region to the tissue space surrounding the target area uncovered by PA signals via model inversion. In ddition, this paper presents a verification method for optical energy deposition in tissues, which observes optical energy at different depths through an energy integration plane. Both the proposed DOPT system and method have been validated through *ex vivo* experiments rather than purely numerical simulations, and they are expected to provide a reference and experimental platform for future research under more complex experimental conditions.

## 2 Theory

### 2.1 Light-Thermal-Acoustic Coupling Mechanism

The mathematical model for energy conversion after light irradiates biological tissue can be represented by the Pennes Bioheat Equation, whose mathematical expression is as follows:

$$\rho c_{p}\frac{\partial T(r,t)}{\partial t}=k_{v}\nabla^{2}T(r,t)+S(r,t)+S_{\mathrm{met}}-\rho_{b}c_{b}\omega_{b}(T-T_{b}),$$ (1)

where ρ, $k_{v}$, and $c_{p}$ represent the density, heat capacity, and thermal conductivity of the tissue, whereas T(r,t) and S(r,t) are the local temperature and PTT heat source of the tissue at position r and time t, respectively. Furthermore, $S_{\mathrm{met}}$ denotes the metabolic heat generation rate of tissue. In PTT, this term is numerically much smaller than S and can be separated from S for independent analysis in the linear model. Therefore, it is neglected in the scenario of this study. The fourth term on the right-hand side of the Eq. (1) describes the influence of blood perfusion on the temperature field within tissue, where $\rho_{b}$, $c_{b}$, and $\omega_{b}$ represent blood density, blood specific heat capacity, and blood perfusion rate, respectively, and $T_{b}$ is the arterial blood temperature. This study mainly focuses on experimental demonstrations using *ex vivo* tissue. Introducing this term would significantly increase the difficulty of modeling and experimental design and is inconsistent with the characteristics of *ex vivo* tissue. Therefore, to simplify the model, this term is temporarily excluded in the current research.

When using laser heating, S(r,t) can be described as:

$$S(r,t)=\mu_{a}(r)\Phi (r,t),$$ (2)

where $\mu_{a}$ is the optical absorption coefficient of the tissue and Φ(r,t) is the local light flux. It can be seen that under photothermal conditions, the distribution of the heat source after tissue heating is related to the optical absorption coefficient and the light flux. Under laser irradiation, the distribution of light flux in the tissue can be described as:

$$\frac{\nabla^{2}\Phi (r,t)}{3(\mu_{a}+\mu_{s}^{\prime })}-\mu_{a}\Phi (r,t)+\frac{1}{c}\frac{\partial \Phi (r,t)}{\partial t}=-L(r,t),$$ (3)

where c is the speed of light, L(r,t) is the equivalent light source, and $\mu_{s}^{\prime }=(1-g)\mu_{s}$ is the reduced scattering coefficient, g is the anisotropy factor. Based on Eqs. (1)–(3), a thermal conduction model of tissue under photothermal effects can be established, completing the creation of a photothermal coupled physical field. When tissue is irradiated with a pulsed laser, if the pulse duration is shorter than the tissue thermal relaxation time, the laser energy will not cause a significant temperature rise in the tissue, but will instead induce changes in internal tissue pressure. This process can be described as:

$$p_{0}(r)=\Gamma (r)\Phi (r)\mu_{a}(r),$$ (4)

where Γ is the Gruenisen coefficient within the tissue. Changes in local sound pressure cause internal stress imbalances in the tissue, which in turn trigger a series of high-frequency vibrations. The mechanical energy from these vibrations will propagate outward in the form of ultrasound,

$$\nabla^{2}p(r,t)-\frac{1}{v_{s}^{2}}\frac{\partial^{2}p(r,t)}{\partial t^{2}}=-p_{0}(r,0),$$ (5)

where p(r,t) is the sound pressure at position r at time t, $v_{s}$ is the sound velocity. The variation of this sound pressure physically manifests as ultrasound, which can be detected by an US transducer located at d and converted into an electrical signal s(d,t). By arranging such transducers in a geometric array within a spatial plane, PA signals from different directions can be collected. Based on these signals, universal back-projection (UBP) algorithms^31^ can be used to restore the local sound pressure distribution. After image reconstruction, the acoustic pressure source at the reconstruction position r, denoted as $p_{0}^{\prime }(r)$, is given by:

$$p_{0}^{\prime }(r)=\frac{2}{\Omega_{0}}\int_{\Omega_{0}}{\left[p^{\prime }(d,t)-t\frac{\partial p^{\prime }(d,t)}{\partial t}\right]}_{t=\frac{\left|r-d\right|}{v_{s}}}d\mathrm{\Omega .}$$ (6)

In the formula, $p^{\prime }(d,t)$ is the signal received by the transducer at position d at time t after undergoing spatial deconvolution and temporal deconvolution, and $\Omega_{0}$ and dΩ represent the total spatial angle of the transducer array and the spatial angle of a single transducer element, respectively. The relationship between the initial sound pressure distribution matrix calculated through image reconstruction and the actual sound pressure matrix satisfies:

$$p_{0}^{\prime }(r)\propto p_{0}(r)\propto \mu_{a}\Phi (r).$$ (7)

By synthesizing the results derived from Eqs. (2), (4), and (7), we conclude that when pulsed and continuous lasers irradiating the tissue surface exhibit identical energy distributions and wavelengths, the spatial patterns of optical energy deposition induced by both laser types within biological tissue remain consistent over the pulse duration t. With the continuous laser operating at a constant energy level, its optical energy deposition in the tissue further reduces to a time-invariant constant. Therefore, the initial acoustic pressure distribution within the tissue is reconstructed before the tissue temperature is elevated by continuous laser irradiation, and the heat source distribution inside the tissue under continuous laser irradiation can be acquired via linear transformation of this matrix. In this paper, we define the aforementioned constraint relationship as matching illumination condition (MIC).

### 2.2 State Estimation and Correction

The PA effect can not only observe the distribution of thermal sources in the target area under photothermal conditions but also has the capability to measure temperature. The intensity of the PA signal has a linear correlation with temperature. Research has shown that this phenomenon originates from the temperature dependence of the Grueneisen parameter. Here, we assume that the Grüneisen coefficient of the same tissue type remains spatially uniform. The dependence of the Grueneisen parameter on temperature T in Eq. (4) can be expressed as:

Γ=A+BT. (8)

In the formula, A and B are constants related to the nature of the tissue. By combining Eq. (8) with Eq. (4), based on the initial temperature $T_{0}$ known before heating, we can define the PA intensity increment operator α:

$$\alpha =\frac{p_{0}(T)-p_{0}(T_{0})}{p_{0}(T_{0})}.$$ (9)

Furthermore, we can calculate the temperature increment $\Delta T=T-T_{0}$ of the target area based on α. We define the spatial position that exhibit a significant PA effect under pulsed laser irradiation as a theoretically observable point (TOP) and arrange the ΔT at all observable points into a column vector ΔT:

$$\Delta T=\frac{A+BT_{0}}{B}\alpha .$$ (10)

Here, α is a column vector formed by arranging the values of α at all TOPs. Under the condition that the Gruneisen coefficient is uniformly distributed, it is linearly related to the ΔT. Equation (10) can be abstracted as a temperature sensor based on the PA effect that can observe the temperature field inside tissues. On this basis, by combining the initial heat source distribution within the tissue to establish a simulation model, temperature prediction and correction can be achieved, and this process can be described by the following Eq. (11).

The dynamic process of temperature changes in the temperature field under the action of the initial heat source S can be expressed using partial differential equations:

$$\Delta T_{i,j,k}^{n+1}=\Delta T_{i,j,k}^{n}+\Delta t[\frac{k_{v}}{\rho c_{p}}\frac{\nabla^{2}(\Delta T_{i,j,k}^{n})}{\Delta l^{2}}+\frac{S_{i,j,k}}{\rho c_{p}}],$$ (11)

where the subscript (i,j,k) denotes the three-dimensional (3D) index within the simulation grid, with each grid having a length of Δl, and the superscript n indicates the physical quantity at the n’th simulation step, or at time nΔt. Equation (11) presents the explicit Euler discretization scheme. To ensure numerical stability, the Δt must satisfy the Courant–Friedrichs–Lewy stability condition, meaning that Δt is jointly constrained by the spatial grid size and the thermal diffusivity of the tissue, as shown in Eq. (12):

$$\Delta t\leq \frac{(\Delta l{)}^{2}\rho c_{p}}{6k_{v}}.$$ (12)

We define the 3D grid as the region of interest (ROI), and the points within it as ROIPs. The set consisting of all ROIPs is denoted as {ROIPs}. Under the condition of neglecting the intrinsic biological thermal metabolism, the source term S originates solely from the external laser heat source. Furthermore, when the illumination condition satisfies MIC, the spatial point set of all S coincides with the set {TOPs}. In general cases, {TOPs} is a subset of {ROIPs}. Equation (11) can be expressed in matrix form:

$$[\begin{array}{c} \Delta {T_{\mathrm{ROI}}}^{n+1}\\ {S_{\mathrm{TOP}}}^{n+1} \end{array}]=F[\begin{array}{c} \Delta {T_{\mathrm{ROI}}}^{n}\\ {S_{\mathrm{TOP}}}^{n} \end{array}],$$ (13)

where the superscripts n and n+1 denote one iteration step of the physical field, whereasthe subscripts TOP and ROI indicate that the vectors are derived from the sets {TOPs} and {ROIPs}, respectively. For instance, $S_{\mathrm{TOP}}$ represents a column vector formed by arranging the scalar values of the heat source at each point within {TOPs}. The F is the system’s state transition matrix, and the system’s state x consists of the ΔT and the S. The theoretical estimated value of the model can be calculated through the system state vector x and the transition matrix F.

The block representation of matrix F is given by:

$$F=[\begin{array}{cc} F_{T} & F_{ST}\\ 0 & F_{S} \end{array}],$$ (14)

where $F_{T}$ is the state transition submatrix for tissue temperature, which mainly describes the heat conduction induced by temperature differences between adjacent grids. The initialization of this submatrix mainly refers to Eq. (11). We will also provide a piece of code in the Supplementary Materials that can be directly used to generate the $F_{T}$ matrix. $F_{\mathrm{ST}}$ is the heat source action submatrix, which describes the temperature variation at points directly heated by the heat source. The initialization of this submatrix only requires matching the heat source with the corresponding temperature nodes, and all nonzero values in the matrix are $\Delta t/(\rho c_{p})$. $F_{S}$ is the state transition submatrix for the heat source itself. A constant-power heat source is used for heating in this study, so $F_{S}$ is an identity matrix. All elements within the F matrix can be formulated and described using tissue-specific parameters; for heterogeneous tissues, only corresponding adjustments need to be made in combination with the tissue parameters at the current location during the initialization of the F matrix.

Further define the set {AOPs} as the set of all actual observable points. Under the ideal conditions of a 3D transducer array and full-view imaging, {AOPs} and {TOPs} are the same set. When in the case of limited sampling perspectives or a two-dimensional (2D) array, {AOPs} is a subset of {TOPs}. Based on Eq. (10), the system’s temperature observation matrix H can be derived:

$$H=[\begin{array}{ccc} \frac{B}{A+BT_{0}}I_{M*M} & 0_{M*(N-M)} & 0_{M*W} \end{array}],$$ (15)

where M, N, and W denotes the number of grid points in the set {AOPs}, {ROIPs}, and {TOPs}, respectively. Under the condition that the Γ is uniformly distributed, the first term of H corresponds to the observable positions with PA signals, the second zero matrix represents the unobservable ROI positions, and the third zero matrix indicates that the $S_{\mathrm{TOP}}$ cannot be directly observed during the iteration. When the Γ within the tissue is nonuniform, or when {AOPs} contains two or more types of tissues with different properties, the first term of the matrix requires more detailed block partitioning to separately calibrate the Γ(r) of different tissue regions. The relevant calibration method can be found in Ref. 32.

Considering both the noisy observations α and the theoretical state x, the Kalman filter can be used to calculate the system’s optimal state $\hat{x}$:

x^n=xn+K(α−Hxn). (16)

In the Eq. (16), K is the Kalman gain. Depending on the extent to which the sensor data α is contaminated by noise, K is continuously updated during the iteration of the temperature field and can be expressed as:

$$P^{n}=F{\hat{P}}^{n-1}F^{T}+Q_{kf},$$ (17)

$$K=P^{n}H^{T}(HP^{n}H^{T}+R{)}^{-1},$$ (18)

P^n=(I−KH)Pn. (19)

In the formula, P and $\hat{P}$ represent the covariance and estimated covariance matrices, respectively, whereas $Q_{kf}$ and R represent the variances of process noise and measurement noise. In engineering practice, the value of $Q_{kf}$ depends on the rate of temperature change: $Q_{kf}=1^{-5}∼1^{-4}$ under steady-state conditions, and $Q_{kf}=1^{-3}∼1^{-2}$ during the heating transient. The selection of R should be combined with the actual system. Signals are acquired in a static, nonheating state for a period, and R is calculated based on the fluctuation level of the collected signals.

## 3 Method

### 3.1 System Design

**Fig. 1:**
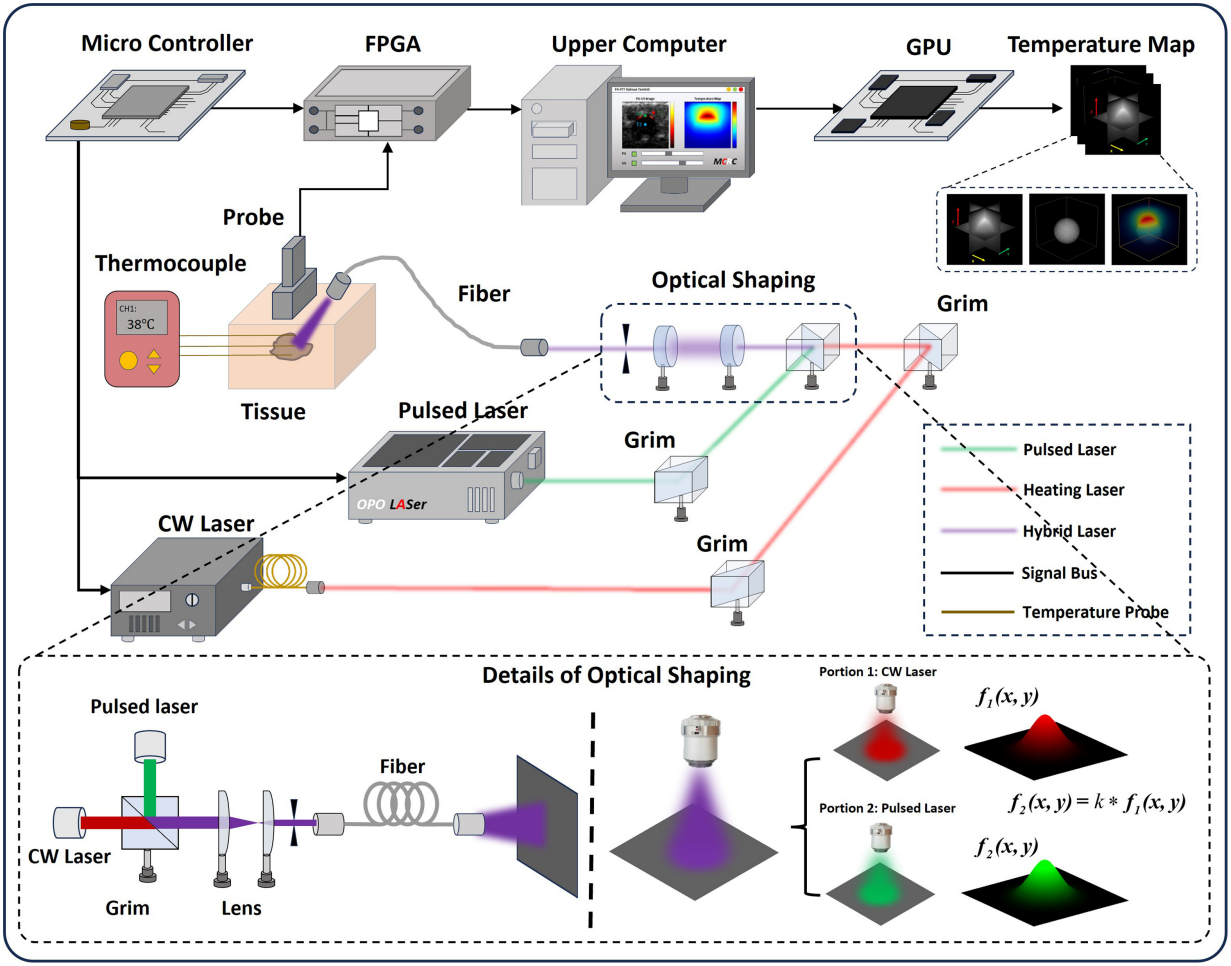
DOPT system diagram: CW laser (LWIRL808-7W-F, Laserwave, China), pulsed laser (SpitLight, Innolas, Germany), thermocouple (UT325F, UNI-T, China), FPGA (Prodigy, S-sharp, China), GPU (NVIDIA geForce RTX 4090 D).

To verify the effectiveness of the proposed method, we constructed a DOPT system as shown in Fig. 1 below. The system is composed of an optical module and an Fig. 1 below. The system is composed of an optical module and an US-PA imaging platform with the linear probe (128 elements and 5.5 M center frequency). The probe is mounted on a three-axis displacement platform to perform tomographic scanning. In addition, we used multichannel thermocouples embedded inside the target sample to collect the actual temperatures at different positions in the region. The optical module includes a pulsed optical path and a heating optical path. The two types of lasers have the same wavelength (808 nm) and form a mixed optical path after beam combining and shaping. Under this design architecture, the light flux distribution generated by the pulsed laser and continuous laser in the tissue and the integral of the converted energy within the duration of the pulsed light are basically consistent. Thus, the initial PA pressure matrix can be used in combination with the tissue light transmission model to observe the internal heat source distribution of the tissue under continuous laser irradiation.

### 3.2 Preoperative Estimation Method

**Fig. 2:**
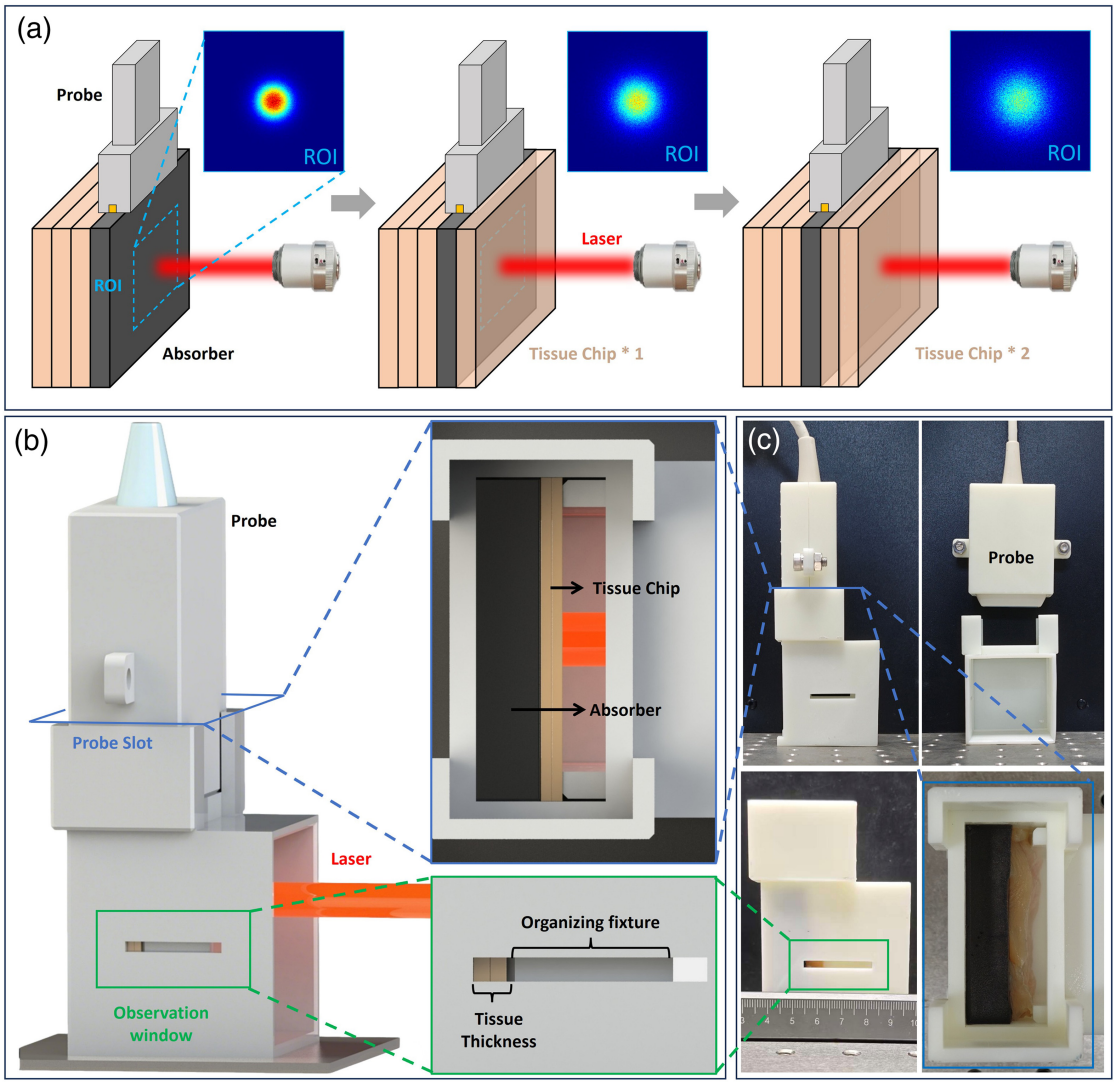
Schematic diagram of the light energy deposition verification device. (a) Schematic diagram of the experimental setup. (b) Model structure and local schematic of the experimental device. (c) Photograph of the actual device.

We propose a method to verify the accuracy of the simulation algorithm for light energy deposition in tissues. Several pieces of chicken breast were taken, cut into thin slices, and stacked to a sufficient thickness. A flat-plate black agarose gel was placed behind the chicken breast to provide sufficient light absorption, forming an energy absorption plane. Spatial light was used to directly irradiate the stacked chicken breast tissue, and an US probe was employed to receive signals. The imaging plane of the probe was ensured to coincide with the energy absorption plane, and the thickness of the chicken breast in the front was increased one by one, as shown in Fig. 2(a). Due to the strong light absorption of black ink, the black agarose gel can be approximately regarded as intercepting all light energy, whereas the remaining energy is distributed in the upper muscle tissue. This method is equivalent to calculating the integral of light energy along the depth. By analyzing the energy integral at different depths, i.e., the intensity of the PA effect, and fitting and comparing it with the simulation results, the accuracy of the predicted light energy deposition can be verified. Such a design can obtain the final light flux distribution and relative intensity of spatial light reaching the agarose light-absorbing plane after attenuation at different propagation depths. Based on the above concept, we designed a light energy attenuation rate measurement device. The main body of the device was designed by 3D printing, which was accurately coupled with the imaging plane and the energy receiving plane in accordance with the physical and geometric dimensions of the US transducer. The specific schematic diagram of the model and the physical photograph are shown in Figs. 2(b) and 2(c).

In the experiment, intact chicken breast tissue was selected and sectioned, and the thickness of the stacked tissue was gradually increased from 2 to 8 mm in 1 mm increments. The tissue slices of varying thicknesses were irradiated with spatial light of 3 mm in diameter and 808 nm in wavelength, and PA signals were acquired. To avoid nonlinear PA effects caused by excessive laser energy, the single-pulse laser energy was set to 1 mJ, with the energy density below the threshold specified in the ANSI standard of $20\;\mathrm{mJ}/{\mathrm{cm}}^{2}$. Detailed simulations and grid parameters are provided in the Supplementary Materials.^33^

### 3.3 Simulated Photothermal Therapy Experiment

Samples for simulating PTT were fabricated from chicken breast and simulated tumors, where chicken breast was used to mimic healthy tissues. The simulated tumors consisted of black agarose spheres. The black ink filled in these spheres significantly enhanced the light absorption capacity of the simulated tumors, which can be used to mimic targeted photothermal probes injected into the body during PTT. These probes specifically refer to biocompatible nanomedicines with strong light absorption properties, which can significantly enhance the photothermal conversion efficiency at the target site and improve the therapeutic efficacy of PTT.^16^^,^^34^ The simulated tumor is set to have a diameter of 5 mm, and its depth from the tissue surface is determined via US imaging. Heating was performed using the DOPT system shown in Fig. 1, and the algorithm proposed in this paper was adopted for simulation and treatment temperature prediction. Meanwhile, multichannel thermocouples were embedded separately in the *ex vivo* tissue. Four channels of the thermocouples were arranged at the tumor upper surface, the tumor core, the lateral tumor surface (outside the imaging plane), and the surrounding chicken breast tissue beyond the tumor, respectively. The thermocouple located the tumor upper surface is within the {AOPs} and is heated up by continuous laser irradiation. Ultimately, through heat conduction, the temperature in the surrounding area rose and was monitored by the other three thermocouples. Four thermocouples were placed at different positions to evaluate the accuracy of the temperature measurement results.

The temperature window required for mild PTT ranges from 42°C to 45°C, whereas the physiological temperature of biological tissues is generally 36°C. To enhance the persuasiveness of this study, simulated PTT experiments on *ex vivo* tissues were performed within the temperature range of 36°C to 45°C. The *ex vivo* tissue samples were placed in a constant-temperature water bath. The calibration procedure for the matrix H is described as follows. After placing the tissue sample in the water bath, the initial temperature was set to 30°C. Once the internal temperature of the tissue became relatively uniform, the corresponding PA signals were acquired. Subsequently, the temperature was increased by 0.5°C, and the above acquisition process was repeated until the temperature reached 36°C. The collected dataset was then used to calibrate the matrix H. This calibration approach avoids thermal damage to the tissue. Following calibration validation using low-power laser irradiation, the formal experiments were initiated.

**Fig. 3:**
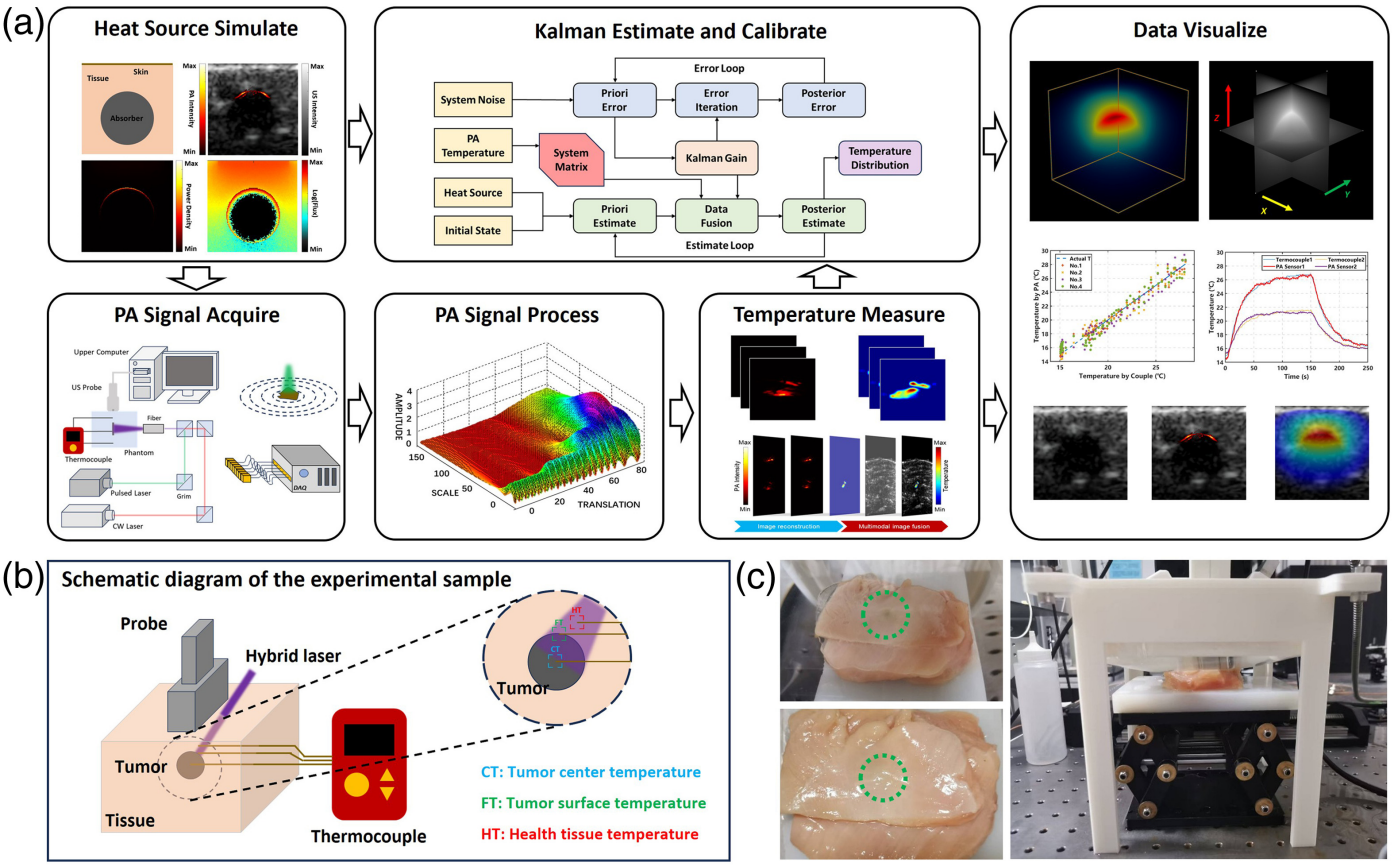
Simulation of PTT experiment. (a) Experimental procedure: light irradiation conditions and heat source simulation, data acquisition, temperature measurement, estimation correction, and visualization. (b) Schematic diagram of the experimental sample setup. (c) Photograph of the sample.

The experimental process is illustrated in Fig. 3(a): after constructing consistent illumination conditions using the DOPT system, pretreatment planning was first conducted. During the pretreatment planning phase, the linear array probe is translated to collect tomographic PA-US multimodal imaging data of the target region. A laser spot with a diameter of 3 mm and a wavelength of 808 nm is adopted throughout this step, with the laser irradiation position kept fixed and the continuous wave laser turned off to prevent any temperature elevation in the tissue. The US component of the tomographic imaging data is utilized to identify tissue structures and initialize the tissue mesh structure. Subsequently, the heat source distribution within the tissue is simulated and reconstructed under MIC conditions. The reconstructed heat source distribution is then spatially registered with the PA component of the tomographic images, and the true 3D heat source distribution within the target region is ultimately determined.

Subsequently, simulated PTT was initiated: the target area temperature measured by the PA thermometry was used as sensor data, and the LTU Model with Kalman filtering algorithm was simultaneously employed to predict the temperature in the tissue. At the same time, the multichannel thermocouples inserted in the sample collected the actual temperatures of different regions in real time to verify the computational accuracy of the proposed method for the temperature in the tissue [Fig. 3(b)]. Figure 3(c) shows a photograph of the sample, where the simulated tumor tissue was embedded in chicken breast tissue and marked with a green dashed box. Detailed simulations parameters are provided in the Supplementary Materials.

## 4 Experiment and Result

### 4.1 Accuracy Verification of Preoperative Estimation Method

**Fig. 4:**
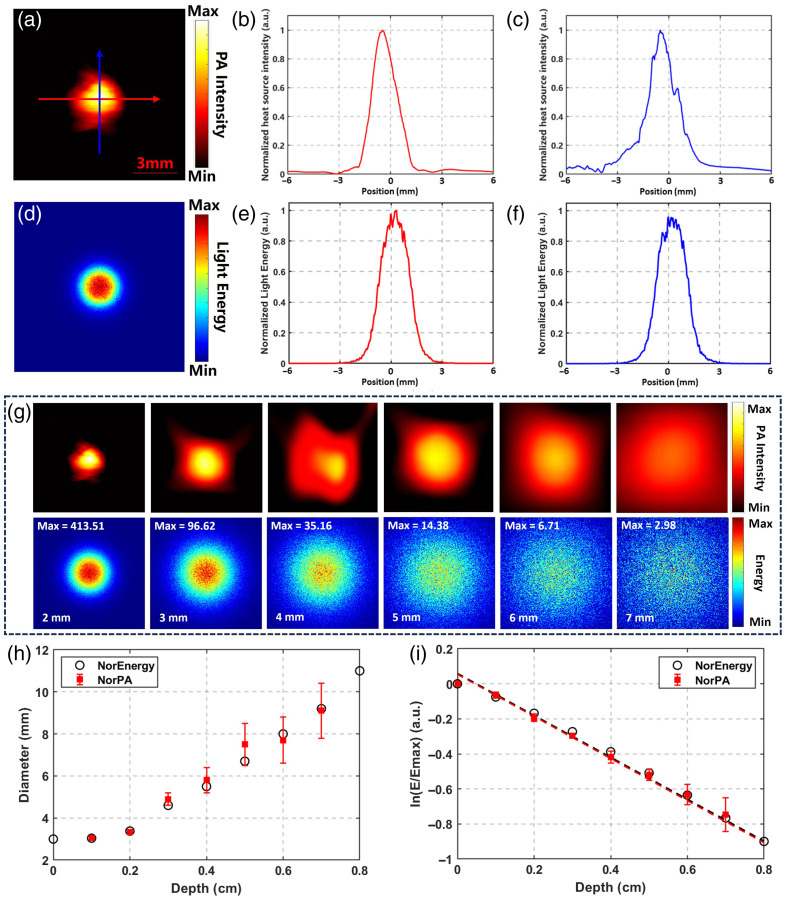
Validation results of intratissue optical energy distribution. (a) PA pressure source distribution acquired using experimental samples. (b) Lateral pressure intensity profile at the center of the pressure source. (c) Axial pressure intensity profile at the center of the pressure source. (d) Simulated optical energy deposition distribution at a depth of 2 mm. (e) Central lateral energy intensity profile. (f) Central axial energy intensity profile. (g) Comparison of PA imaging results and simulation results at different penetration depths. (h) Comparison of fitted spot diameter at different depths. (i) Fitted results of energy deposition calculation at different depths.

According to the proposed verification method for light energy deposition, the thickness of chicken breast above the energy absorption plane was varied, and an US probe was used to receive PA signals and reconstruct PA images. Figure 4(a) below shows the reconstructed initial acoustic pressure distribution image at a penetration depth of 2 mm. The acoustic pressure intensity directly depends on the integral of light energy deposition at local positions. It can be intuitively observed from the image that the geometric characteristic of the integral of energy deposition at any depth after spatial light irradiating the tissue on the plane is an approximate circular Gaussian spot. A threshold of 5% of the maximum acoustic pressure intensity was used to filter out imaging artifacts and low-frequency noise interference, determine the spot boundary, and the geometric center of the spot was obtained through fitting. Taking this geometric center as the origin, PA intensity profiles were plotted along the horizontal direction [Fig. 4(b)] and vertical direction [Fig. 4(c)]. The profile curves indicate that the energy intensity attenuates with increasing radius relative to the light irradiation center, with the full width at half maximum (FWHM) in the horizontal and vertical directions being 0.172 and 0.166 cm, respectively.

The optical simulation algorithm is primarily based on Monte Carlo modeling. In the simulation code, a 3D spatial grid is constructed to represent the tissue, and each voxel is assigned optical absorption and scattering coefficients that mimic the properties of chicken breast tissue. To simulate the agarose gel phantom inserted at various depths in the actual experiments, the optical properties of local tissue at specific depths within the grid were modified accordingly, with the depths aligned to the experimental setup. Simultaneously, the illumination conditions were defined on the upper surface of the simulated tissue, featuring a spot size of 3 mm that matches the optical parameters of the system. Figure 4(d) shows the simulated energy integral distribution image at 2 mm. The integral intensity curves in the horizontal direction [Fig. 4(e)] and vertical direction [Fig. 4(f)] also present Gaussian-like curves. The simulated energy distribution is an almost standard circle with an FWHM of 0.167 cm. Comparing the FWHMs of the simulated data and experimental data, the errors in the horizontal and vertical directions are 2.99% and 0.60%, respectively. This result demonstrates the accuracy of the simulated results in terms of spatial morphology. By varying the thickness of chicken breast, the visual comparison between the actual energy distribution and simulated results at different depths is shown in Fig. 4(g). Four chicken breast tissue slices were prepared and tested to meet statistical requirements. The spot contours were extracted, fitted, and their diameters were calculated. The comparison between the simulated results and actual results at different depths is presented in Fig. 4(h), with both errors within a controllable range.

In addition to morphological distribution in geometric space, this study validated the relative intensity of light energy deposition at varying penetration depths. Relative energy intensity was normalized to the intensity at zero penetration depth, yielding values theoretically ≤1. Absolute energy at each depth was derived by integrating the energy absorption plane, and relative energy was obtained by ratioing to the energy at zero depth. Simulation data (NorEnergy) and experimental data (NorPA) were compared. Log-compressed results of both datasets were highly linearly correlated with increasing depth ($R^{2}=0.998$), confirming that cumulative energy intensity decays exponentially, with a discrepancy <3%. Fitting results are displayed in Fig. 4(i). The measured data were scaled by a factor of $3.04\times 10^{-3}$ for quantitative consistency with normalized simulation results. This amplitude difference is attributed mainly to systematic errors including acoustic-electric conversion gain, optical coupling loss, and absolute intensity calibration. These findings provide a basis for follow-up investigations.

### 4.2 Result of Simulated Photothermal Therapy

**Fig. 5:**
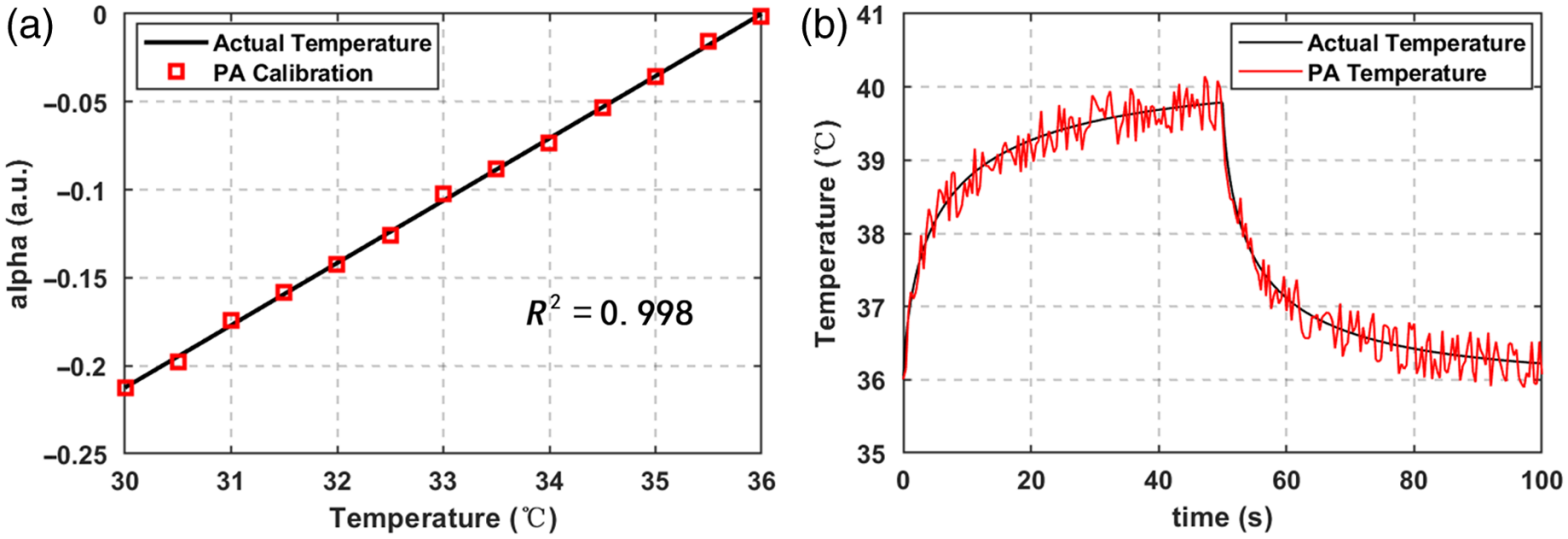
Calibration results of matrix H. (a) Fitting results of α versus temperature in low-temperature calibration; (b) test results under low-power heating.

The matrix H was calculated according to the experimental procedures in Sec. 3.3 and Eq. (8)–10. The PA enhancement factor α was fitted against the actual temperature in the constant-temperature water bath, and the coefficient of the identity matrix term (the first entry) of the H matrix was finally determined to be 0.0354. Figure 5(a) shows the fitting relationship between α and the actual temperature, with a linear correlation coefficient of 0.997.

Based on the calibrated sensor parameters, the sample was irradiated with low laser power heating to verify the dynamic response characteristics of the system. The dynamic temperature tracking curves of the two heating processes are shown in Fig. 5(b). The reference temperature curve output by the thermocouple attached to the upper surface of the simulated tumor and the temperature curve output by the PA thermometry method remained highly consistent during the 50 s heating process and 50 s cooling process, with a maximum temperature measurement error of 0.39°C throughout the process. This result demonstrates that the sensor based on the PA effect enables real-time and accurate temperature measurement in the target region.

The sample was cooled down to 36°C, and the relative position between the sample and the optical path was maintained constant. The position of the probe was adjusted to carry out tomographic scanning. The structural parameters of the target region were identified via the US component, whereas the heat source of the target region was reconstructed using the PA component. The optical energy deposition simulation was implemented based on the reconstructed structural parameters of the target region.

**Fig. 6:**
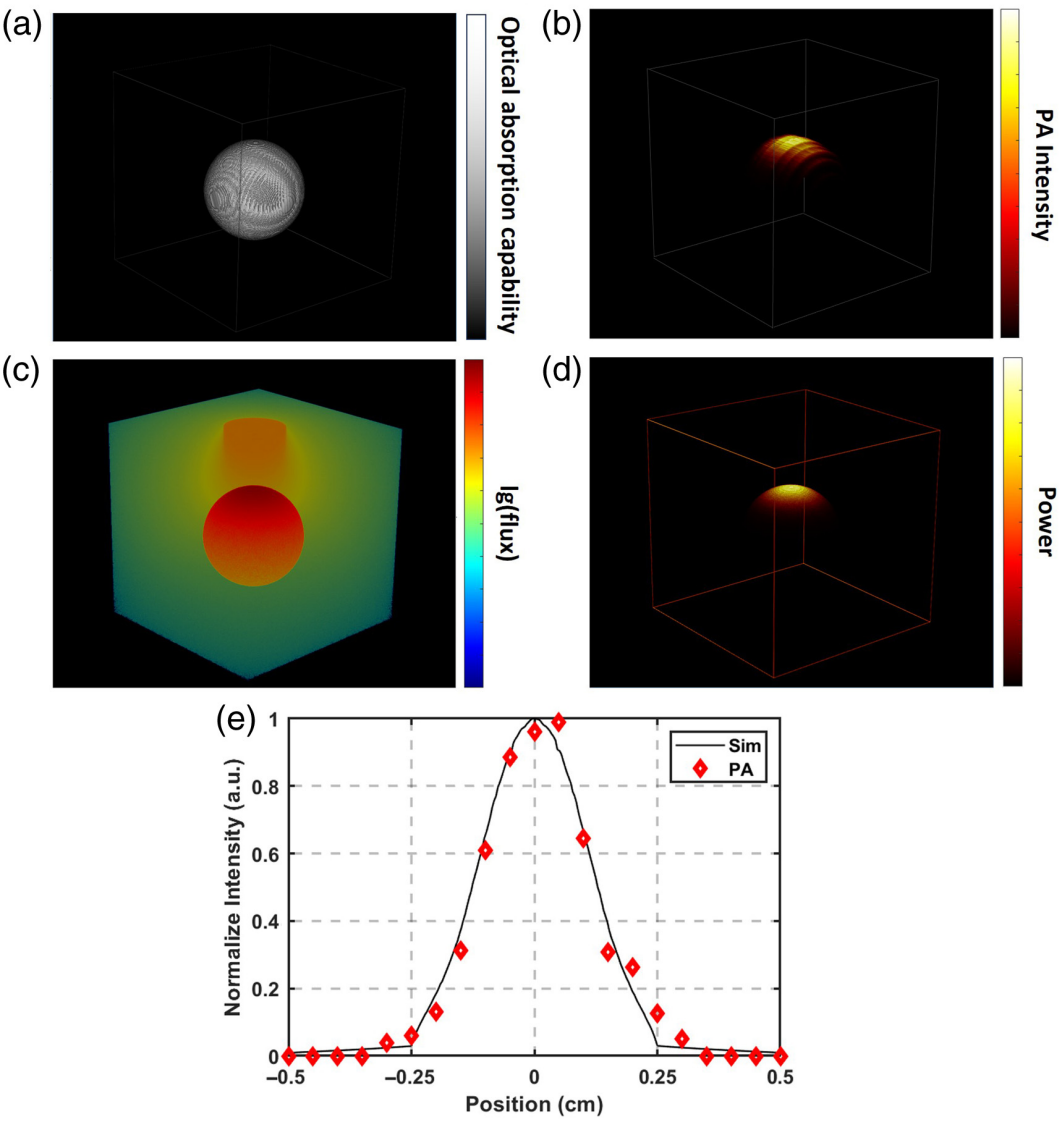
Results of target region reconstruction for 3D heat source in preoperative planning. (a) 3D structural display of the target region mesh. (b) Heat source distribution reconstructed based on the PA component. (c) Optical flux distribution in the target region (simulation). (d) Heat source distribution in the target region (simulation). (e) Comparison of numerical intensity profiles between the imaging section and the simulation section.

Figure 6(a) presents the regional mesh extracted from the US image of the target region based on the structural parameters identified by the US component. The upper surface of the simulated tumor is approximately 3.2 mm away from the tissue surface. Figure 6(b) shows the 3D structure reconstructed based on the PA component, where the region with PA effect is located on the upper surface of the simulated tumor. Figures 6(c) and 6(d) illustrate the simulation results, namely, the 3D visualization of luminous flux in the target region and the 3D visualization of the heat source, respectively. Because light undergoes an exponential decay in tissues, logarithmic compression was applied to the luminous flux mesh to facilitate visualization. A comparative analysis of Figs. 6(b) and 6(d) demonstrates that both the simulated heat source and the experimentally acquired 3D PA results are distributed on the upper surface of the simulated tumor.

Figure 6(e) presents the detailed quantitative analysis results of the simulation results and actual data. The simulated heat source mesh was sectioned along the scanning direction of the actual system, with the section positions matched with those of the multimodal imaging results. The heat source values and PA pressure values within the region were extracted and subjected to normalization fitting. As shown in the figure, the intensity profiles of the two are basically consistent. The horizontal axis of Fig. 6(e) was subdivided into infinitesimal elements to calculate the integral area including the envelope profile, and the error between the two integral values was 3.5%. Potential causes of the error include noise interference in the actual system, which masks the weak-intensity signal regions on both sides of the upper semicircle and prevents their accurate restoration in the PA image. In addition, the limited sampling angle of the linear array probe adopted in this study also leads to the loss of partial lateral information. However, these errors do not have a significant impact on the target region temperature assessment during the treatment process. Therefore, the actual heat source mesh can be filled by combining the simulation results to recover more heat source details.

**Fig. 7:**
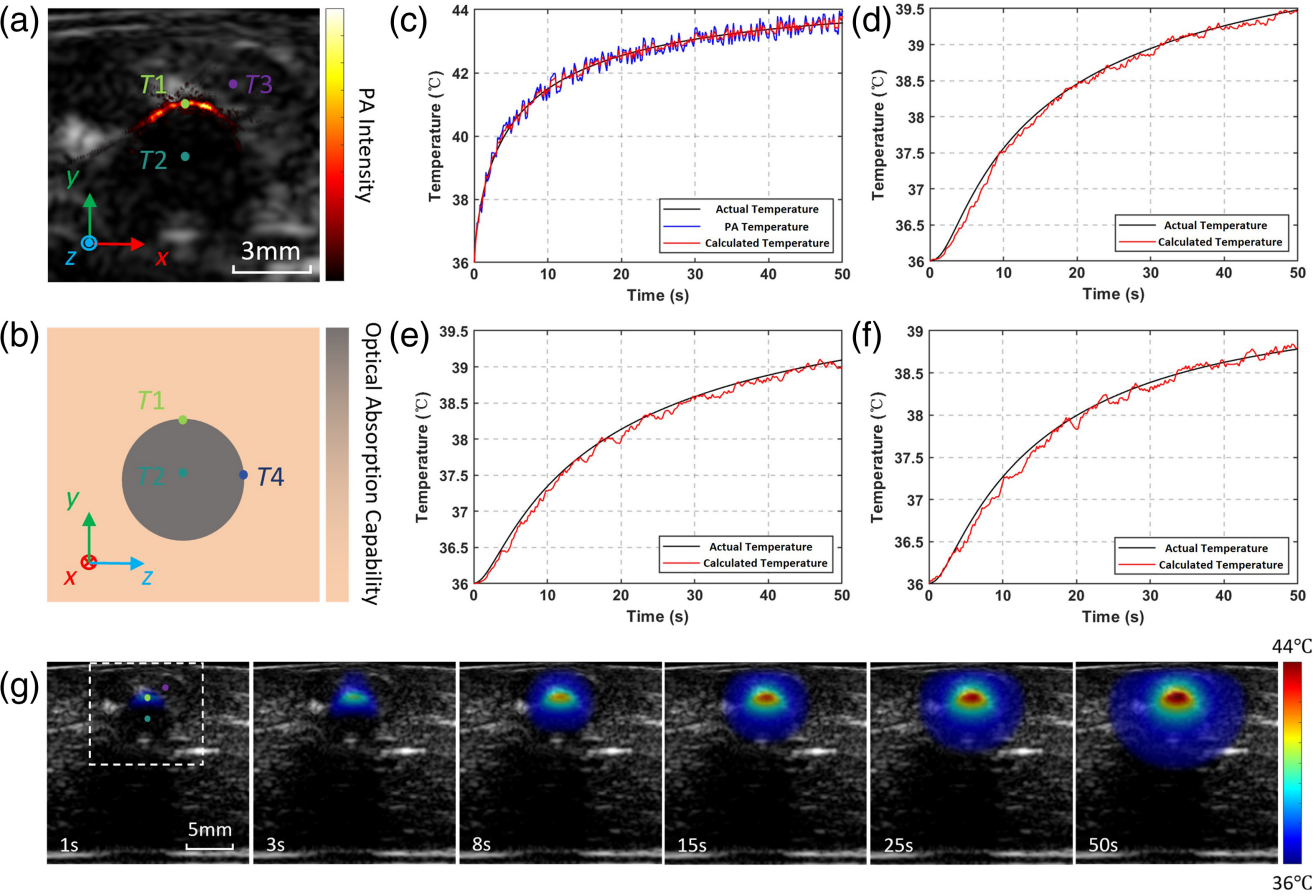
Results of PTT experiment. (a) PA-US multimodal image and positions of thermocouple sampling sites. (b) Schematic diagram of sampling site positions outside the imaging plane. (c) Temperature curve T1 of the directly heated area on the tumor surface. (d) Temperature curve T2 of the tumor core area. (e) Temperature curve T3 of the surrounding healthy tissue. (f) Temperature curve T4 of the tumor margin. (g) US-Temperature dual-modality imaging results of the target area during heating.

Figure 7 illustrates the results of simulated PTT. Figure 7(a) presents the US-PA dual-modal image of the tissue region, where the key sampling positions of three thermocouples are explicitly labeled. These sampling sites correspond to the temperature at the upper boundary of the tumor (T1), the temperature at the tumor core (T2), and the temperature of the adjacent healthy tissue (T3), respectively. The schematic diagram of the lateral cross-section is depicted in Fig. 7(b). This cross-section is perpendicular to the imaging plane, with the intersection line of the two planes coinciding with the central axis along the y-direction of the imaging plane. The sampling point T4 is situated on this cross-section; due to the inability of the probe to perform rotational image acquisition, this position is only presented in the form of a schematic illustration.

Figures 7(c)–7(f) depict the temperature-time curves of the four sampling points (T1, T2, T3, and T4). In these figures, “Actual Temperature” refers to the temperature values directly measured by the thermocouples, while “Calculated Temperature” denotes the output temperature calculated by the method presented in this article. It is noteworthy that the T1 region exhibits PA signals and thus falls within the observable range of the PA thermometry method. Consequently, the temperature measurement results obtained by PA thermometry are additionally included in Fig. 7(c).

The maximum temperature measurement error of the PA thermometry method is ±0.39°C, which is consistent with the conclusions derived from the calibration and verification experiments described in the preceding sections. The primary source of this error stems from the random energy fluctuation of the pulsed laser, which induces variations in the α factor. During the temperature measurement process, such fluctuations can be classified as random noise, which is within the processing range of the Kalman filtering algorithm. Therefore, by fusing the model output with the PA temperature measurement data via Kalman filtering, the temperature measurement error is suppressed to 0.25°C.

The temperature values at the remaining three sampling points (T2, T3, T4) are obtained through inverse extrapolation based on the thermal conduction laws incorporated in the numerical model. Owing to the influence of process noise during the model establishment, the direct calculation of the temperature using the original model may lead to deviations between the model outputs and the actual physical system. The introduction of Kalman filtering effectively resolves this issue, as the model outputs can be recursively corrected using the PA temperature measurement data. As shown in Figs. 7(d)–7(f), the “Calculated Temperature” curves maintain a high degree of consistency with the “Actual Temperature” curves throughout the entire heating process, with the maximum temperature measurement error also less than 0.25°C. Compared with the “Actual Temperature” in Fig. 7(c), the temperature variations in these three figures are gentler. This phenomenon is primarily attributed to the fact that the thermal conduction process is inherently a mathematical model with large inertia, which acts as a low-pass filter in the frequency domain and thereby further filters out the random noise during the temperature measurement process.

As shown in the above results, the traditional PA thermometry relies on the PA signals in the temperature measurement region. However, due to thermal diffusion and conduction effects, the region exhibiting PA signals in the tissue is much smaller than the high-temperature zone of the internal temperature field. Therefore, this paper instead uses PA signals to observe the heat source region of the tissue through the DOPT system. When the heat source is known, the temperature estimation model can be used to directly calculate the temperature around the target region. Nevertheless, real-world systems are subject to various disturbances and observation noise, and the tissue temperature estimation model may have unmodeled dynamics. Thus, when temperature estimation relies entirely on model derivation, these errors will accumulate gradually, eventually leading to a significant discrepancy between the temperature predicted by the model and the actual temperature.

The introduction of the Kalman filter effectively addresses the limitations of the above two independent schemes, integrating them into a whole. In this whole, the systematic biases caused by imperfect modeling can be treated as process noise, and uncertainties such as environmental disturbances and instrument noise can be suppressed as random noise. Through the iterative optimization of the filter, the deviations in heat source estimation gradually converge, and the model predictions and PA observations correct each other. This not only ensures the temperature measurement accuracy in {AOPs}, but also brings the surrounding temperature inferred by the model closer to the actual value, avoiding problems such as measurement blind spots or error accumulation common in single methods, and fully exerting the complementary advantages of model derivation and PA sensing.

Within the framework of the DOPT system, the ultimate temperature measurement depth of the proposed method depends on the trade-off between the maximum penetration depth of PA imaging and the signal-to-noise ratio. Different laser wavelengths exhibit distinct penetration depths in biological tissues. The combination of multiwavelength lasers and high-performance photothermal nanomaterials can further improve the limiting temperature measurement depth. The analysis method for temperature measurement depth is provided in the Supplementary Materials.

## 5 Discussion

In this study, a unique dual-optical-path configuration is employed to tightly couple the two physical processes of PAI and PTT, through which an intratissue LTU model and a DOPT system are constructed. By integrating theoretical model derivation with empirical system data, the effective temperature measurement range of PA thermometry is substantially expanded. Multiple temperature data from different locations derived from the DOPT system were compared with the sampling values collected from multichannel thermocouples positioned within the target region, and experimental results verify that the temperature measurement errors at multiple typical sampling points in both the target region and surrounding tissues are below 0.25°C under the conditions of *ex vivo* tissue samples and spherical heat sources.

Notably, the current experimental validation is still restricted to linear transducer arrays and 2D imaging planes. In this research, tomographic scanning combined with an optical propagation model is adopted to pre-estimate the distribution of spherical heat sources within the target region prior to treatment. While this protocol exhibits favorable operability in basic experimental verification, it deviates considerably from real clinical scenarios. Deep-seated human tumors generally present irregular 3D morphologies, and photothermal heat sources may shift away from the preset plane due to variations in optical fiber placement; under these circumstances, heat source distribution reconstruction via linear array transducers is prone to induce significant measurement errors. To address this limitation, 2D planar arrays or hemispherical bowl-shaped transducer arrays will be introduced in the next phase of research, which will facilitate 3D PA structure reconstruction of tumors via 2D or 3D sensor arrays, enabling acquisition of more precise heat source distribution data and 3D temperature monitoring throughout the PTT. In addition, the impact of tissue blood perfusion on the temperature field was not taken into account in *ex vivo* experiments. The incorporation of blood perfusion effects necessitates more refined mathematical modeling of biological tissues.

However, despite the aforementioned limitations, the system concept and theoretical model proposed in this work still provide valuable references for future research on temperature monitoring within biological tissues under more complex experimental conditions.

## 6 Conclusion

This study proposes a noninvasive temperature monitoring method in tissues based on the dedicated DOPT system. By performing ternary simulation of light transmission-thermal conduction-acoustic propagation to predict the temperature of the target area, and combining theoretical simulation with the actual system through Kalman filtering and state estimation, noninvasive precision monitoring of the temperature in tissues heated by laser is achieved. On this basis, a DOPT system is designed, and the feasibility of the method is verified through *ex vivo* tissue experiments. Experimental results indicate that the deviations between the data obtained from multiple typical sampling points within the *ex vivo* tissue and the actual measured temperatures are less than 0.25°C.

## Supplementary Material

10.1117/1.JBO.31.5.057001.s01

## Data Availability

The data and code that support the findings during the current study are available from the corresponding author upon reasonable request.
