# Supplementary material for: Noninvasive detection and prediction method for temperature in ex vivo biological tissue based on opto-thermal-acoustic co-coupling
Source: J Biomed Opt. 2026 May 28;31(5):057001. doi: 10.1117/1.JBO.31.5.057001 (PMC13218832; doi:10.1117/1.JBO.31.5.057001)
Supplement: Supplementary file 1 [file JBO_031_057001_SD001.docx]

**Supplementary Materials**

**Supplement to Section 3.2**

This section presents the parameters for optical energy deposition simulation. Considering the effective propagation distance of light, the grid adopted herein is not completely consistent with that used in the photothermal therapy simulation, and it can be mapped to the photothermal therapy grid via coordinate matching.

**Initialized Parament:**

| **Simulation parameter** | |
| --- | --- |
| ROI length (cm) ^1^ | L = 3; |
| Grid density (a.u.) | Nx = 256;  Ny = 256;  Nz = 256; |
| Photon number (a.u.) ^2^ | N = 1e9; |
| **Tissue optical parameter**^3^ | |
| u_a_ (cm^-1^) | 0.08; |
| u_s_’ (cm^-1^) | 1.9; |
| g (a.u.) | 0.9; |

**Boundary conditions:**

Photons escaping from the computational grid will be terminated and no longer involved in subsequent calculations.

1 The ROI defaults to a cube.

2 Light energy simulation serves as a preoperative planning step without real-time requirements, allowing for an increased number of photons as needed.

3 Cited from Ref. [33] in the main text.

**Supplement to Section 3.3**

The simulation parameters for photothermal therapy are presented below. The initialization of the heat source depends on imaging data and optical energy deposition simulation.

**Initialized Parament:**

| **Simulation grid parameter** | |
| --- | --- |
| ROI length (cm) ^1^ | L = 3; |
| Grid density (a.u.) ^2^ | Nx = 128;  Ny = 128;  Nz = 128; |
| Sim step | Base on Eq. 12 |
| **Parameters of chicken breast muscle** | |
| Density (kg/m^3^) | 1040 |
| heat capacity ( J/(kg⋅K) ) | 3500 |
| Thermal conductivity ( W/(m⋅K) ) | 0.51 |
| **Parameters of sim tumor** | |
| Density (kg/m^3^) | 998 |
| heat capacity ( J/(kg⋅K) ) | 4000 |
| Thermal conductivity ( W/(m⋅K) ) | 0.57 |

**Boundary conditions:**

The temperature outside the computational grid is maintained at a constant of 36 °C to simulate the experimental ambient condition, where the sample is immersed in a constant-temperature water bath. When expressed in terms of temperature increment, the temperature increment outside the grid is set to 0 °C.

1 The ROI defaults to a cube.

2 Grid density can be tailored based on GPU hardware resources.

**Supplement to Section 4.2**

This section mainly describes the analytical method for characterizing the ultimate temperature detection depth of the current DOPT system.

Figure S1 presents the variation of the photoacoustic signal intensity of ICG with penetration depth at the wavelength of 808 nm, and the experiments were performed on ex vivo biological tissues. As shown in Fig. S1 (a), photoacoustic signals can still be detected at depths greater than 2 cm. Nevertheless, according to the signal attenuation curve in Fig. S1 (b), the signal intensity decays to 10% of the initial value at a depth of 1.5 cm.

In this study, the overall temperature elevation ranges from 36 °C to 43 °C, corresponding to a total temperature rise of 7 °C. Based on the linear theoretical model, the effective temperature increase contributed by the heat source decreases to 0.7 °C at the depth of 1.5 cm. The systematic disturbance noise is approximately 0.5 °C (see the calibration of matrix ***H*** in the main text). Under this condition, the real temperature rising trend will be submerged by background noise.

The evaluated depth is strongly correlated with system performance and treatment protocols, rather than being a fixed constant. According to the above analysis, the ultimate temperature measurement depth of the proposed method can be optimized through the following strategies:

(1) Employ high-performance photothermal probes to improve the photothermal conversion efficiency;

(2) Suppress systematic noise and reduce the inherent background noise of the system by introducing energy observation constraints;

(3) Optimize laser wavelength selection: adopt lasers with lower attenuation in biological tissues, matched with dedicated photothermal probes;

(4) Increase the laser power within the biosafety permissible range to enhance thermal excitation.


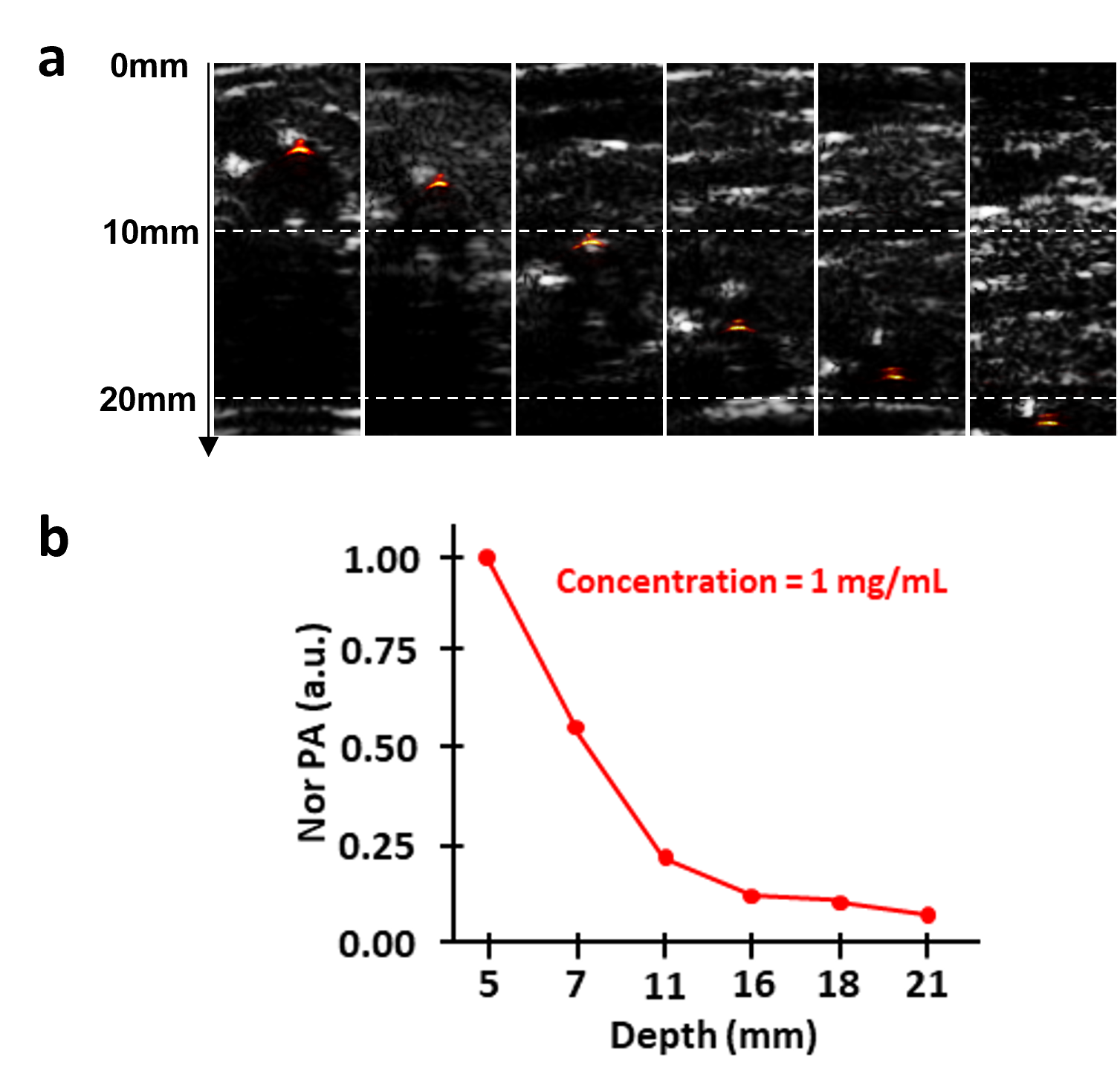


Fig. S1. Quantification of PA imaging depth.

(a) Imaging performance of ICG at different depths.

(b) PA signal intensity of ICG at different depths.

**Supplement to Section 2.2**

Kalman filter system matrix initialization code.

When hardware resources are insufficient, the proportion of the Kalman region in the entire simulation grid can be appropriately reduced; for example, the Kalman matrix is constructed only around the heat source region. Meanwhile, the observable regions in the observation matrix ***H*** can be planned in blocks, which can significantly reduce the computational load of matrix multiplication. For specific details, refer to Eq 15. in the main text. The matrix arranged in this manner can simplify matrix multiplication into matrix block slicing.

***F*_TT_ Initialization Code：**

Matlab

| %%  global Nx Ny Nz;    %% Custom parameters  L = 0.03; % Grid length  dt = 1; % Simulation time step    Nx = 128; % Grid number in the x-direction  Ny = Nx;  Nz = Nx;    rho = 1000; % Density  cp = 3500; % Specific heat capacity  kv = 0.5; % Thermal conductivity    %% End of parameter section  dx = L/Nx;    rc = rho*cp;  kc = kv/dx^2;    Nf = Nx * Ny * Nz;    F_TT = sparse(Nf, Nf);    for k = 1:Nz    disp(k);    for j = 1:Ny  for i = 1:Nx  marg_num = 0;  idx = indx_trs(i, j, k);    if i > 1  marg_num = marg_num + 1;  F_TT( idx, indx_trs(i-1, j, k) ) = 1;  end    if i < Nx  marg_num = marg_num + 1;  F_TT( idx, indx_trs(i+1, j, k) ) = 1;  end    if j > 1  marg_num = marg_num + 1;  F_TT( idx, indx_trs(i, j-1, k) ) = 1;  end    if j < Ny  marg_num = marg_num + 1;  F_TT( idx, indx_trs(i, j+1, k) ) = 1;  end    if k > 1  marg_num = marg_num + 1;  F_TT( idx, indx_trs(i, j, k-1) ) = 1;  end    if k < Nz  marg_num = marg_num + 1;  F_TT( idx, indx_trs(i, j, k+1) ) = 1;  end    F_TT( idx, idx ) = -6;    end  end  end    F_TT = speye(Nf) + dt*kc/rc*F_TT;    function indx = indx_trs(i,j,k)  global Nx;  global Ny;  global Nz;  indx = (k-1)*Nx*Ny + (j-1)*Nx + i;  end |
| --- |
